# Healthy ageing in Europe: prioritizing interventions to improve health literacy

**DOI:** 10.1186/s13104-016-2056-9

**Published:** 2016-05-12

**Authors:** Julii Brainard, Yoon Loke, Charlotte Salter, Tamás Koós, Péter Csizmadia, Alexandra Makai, Boróka Gács, Mária Szepes, On behalf of the Irohla Consortium

**Affiliations:** Norwich Medical School, University of East Anglia, Norwich, NR4 7TJ UK; Hungarian National Institute for Health Development, Budapest, 1437 Hungary; Faculty of Health Sciences, Doctoral School of Health Sciences, University of Pécs, Pécs, 7621 Hungary; Department of Behavioural Sciences, University of Pécs Medical School, Pécs, 7624 Hungary; Institute of Behavioural Sciences, Semmelweis University Budapest, Budapest, 1089 Hungary; Led by The University Medical Center Groningen, 9700 RB Groningen, The Netherlands

**Keywords:** Health literacy, Older adults, Multi-criteria decision aid, Prioritisation exercise

## Abstract

**Background:**

Health literacy (HL) is low for 40–50 % of the population in developed nations, and is strongly linked to many undesirable health outcomes. Older adults are particularly at risk. The intervention research on health literacy in ageing populations project systematically created a large inventory of HL interventions targeting adults age 50+ , to support practical production of policy and practice guidelines for promoting health literacy in European populations.

**Methods:**

We comprehensively surveyed international scientific literature, grey literature and other sources (published 2003+) for implemented HL interventions that involved older adults. Studies were screened for eligibility criteria and further selected for aspects important in European public health policy, including priority diseases, risk factors and vulnerable target groups. Interventions were prioritised using a multiple criteria tool to select final interventions that also featured strong evidence of efficacy and a broad range of strategies.

**Results:**

From nearly 7000 written summaries, 1097 met inclusion criteria, of which 233 were chosen for scoring and ranking. Of these, seven had the highest multi-criteria scores. Eight more articles were selected based on rounded criteria including a high multi-criteria score as well as elements of innovation. Final selections were 18 articles describing 15 programmes, which feature strong evidence of efficacy among important diseases or risk factors and vulnerable groups, or that had success with elements of innovation were identified. Most programmes tried to increase skills in communication, self-management and understanding healthcare or lifestyle choices.

**Conclusions:**

These programmes have multiple positive attributes which could be used as guidance for developing innovative intervention programmes to trial on European older adults. They provide evidence of efficacy in addressing high priority diseases and risk factors.

**Electronic supplementary material:**

The online version of this article (doi:10.1186/s13104-016-2056-9) contains supplementary material, which is available to authorized users.

## Background

Health literacy (HL) may be defined as “the degree to which people are able to access, understand, appraise, and communicate information to engage with the demands of different health contexts to promote and maintain health across the life-course” [1]. Other definitions of HL and practical intervention models have been presented [2–6], but the largest body of research follows Nutbeam [7], who proposed three sequential levels: functional HL (meaning the most basic literacy and numeracy skills applied to health matters), interactive HL (emphasis on communication skills with and use of information sources), and critical health literacy (which includes evaluation of risks and benefits).

Health literacy is considered relatively low for a high percentage (40–50 %) of the population in developed nations [8–10]. There are strong links between poor health literacy and undesirable impacts on a wide-range of health indicators and outcomes [11]. Low HL may impose additional costs of 3–5 % onto total national health care budgets [12]. In recent years European public health bodies have repeatedly called for coordinated approaches to addressing health literacy issues in the European Union (EU) [13, 14], and several EU-wide surveys have been commissioned to gauge HL deficits [5, 15]. Older adults tend to have especially low health literacy [16], and people over 65 may be considered in their entirety as a vulnerable group.

The Intervention Research on Health Literacy in Ageing populations (Irohla) project was funded for 3 years by the European Union (EU) as part of the seventh framework programme. Many (thousands of) peer-reviewed articles have described health literacy interventions that include older adults. Irohla’s ultimate aim was to identify, test and recommend from this large group of studies a maximum of twenty interventions for improving health literacy in older adults (age 50+), as part of larger EU-wide initiatives to promote healthy ageing. Conducting a systematic search and creating a comprehensive inventory was an important methodological task in Irohla, from which a shortlist of promising interventions could later be recommended for adaptation, piloting and implementation within European countries. Interventions were searched for in health care settings or delivered by health care professionals, as reported in scientific or grey literature or in other sources, and which included older adults (age 50+). We developed and applied a multi-criteria selection tool to identify fifteen interventions with the best balance of priority targeting, good quality design, efficacy and innovation (i.e., novel strategies or approaches for enhancing HL), and recommended these programmes for subsequent development and practical implementation in EU countries.

## Methods

### Conceptual grounding

The phrase and concept of “health literacy” is most established among English- and Spanish-speaking professionals. Irohla [17] conceptualised how the HL concept might be articulated in other languages and cultures, and also identified relatively unusual and hence innovative strategies in previous HL research. Furthermore, the Irohla intervention model of health literacy (IIMHL; [17, 18]) strove to balance the situated nature of the individual older person (and family) in their broader social, economic and cultural context with those of the health professional within the health system. Thus, our search was not merely for studies that used the words “health literacy” but rather for health promotion efforts that had any element of the IIMHL, including patient education about health, informed decision making, skills acquisition, strengthening relevant social networks, removing barriers or efforts to increase patient empowerment at the system level.

The IIMHL itself is underpinned by a detailed taxonomy of health literacy that includes seven objectives classified as modifiable determinants applicable at the individual or system level such as: enablement through information, education and skills development; behavioural, social and contextual support; professional development; and, environmental change or enhancement (see Additional file 1: Table S1 and [19]. The systematic survey reported here is the application of the Irohla model to identify relevant intervention reports, followed by mixed selection methods to identify the very best interventions to form the most promising basis for creating future health literacy promotion programmes in the diverse nations of the European continent.

### Data sources and search methods

The search involved keyword searches (Table 1) within these scientific databases: OVID (part of the Medline group of literature archives), CINAHL and Scopus. The search of scientific databases took place in September 2013. Supplemental searches of specific scientific databases, expert suggestions and grey literature on the Internet were undertaken from September 2013 until 31 Jan 2014. Specific databases for clinical trials, health promotion programmes and previous evidence-based reviews were searched (see list Additional file 1: Box S2). Stakeholders and expert contacts were asked for details about active intervention programmes with a custom-designed online form. Grey literature was searched using the Google advanced search engine (Additional file 1: Box S3). The Internet/grey literature search was duplicated in Dutch, Greek, Italian, Polish, Swedish, Finnish, Spanish and French and German. Results from the first three pages of each Google search return were screened for relevant interventions. There was also a specific English-language search for health literacy interventions among disadvantaged European ethnic minorities within OVID, Google open search (screened first three pages of results only) and Google Scholar using the phrase:

(“Ethnic minority” OR Asian OR Moroccan OR Turkish OR Roma OR Gypsies) AND (European OR Europe OR European-country name) AND (health promotion).

**Table 1:** Terms used to search scientific databases

Outcome domains	Activity type	Attributes	Terms for target groups
Health literacy	Strategies	Skills	Older adults
Numeracy	Program*	Awareness	Seniors
Self-management in health	Campaigns	Knowledge	Pensioner
Health-information seeking	Intervent*		Aging population
Prevent*	Activities		Elderly
Healthy behav*	Examples		Aged
Health promot*	Pract*		Family
Reading	Innovat*		Community
Writing and calculation	Health promot*		Geriatric
	Prevent*		

One match with at least one term in each column, search in all multi-purpose (mp) fields in the search database

* Wildcard expansion

Both scientific and supplemental searches found many review and policy documents that cited evidence from multiple interventions. Most of the review or policy documents, especially if they had specific keywords such as “older adults” (see full list of keywords in Additional file 1: Box S4), were also hand-searched for further relevant intervention studies (a process known as “snowballing”). Items were included after duplicate screening of title and abstract (or other summary information) if they seemed to feature health literacy strategies and objectives as defined by Irohla Consortium [17], and these inclusion criteria:

- Any European language, but abstract or summary available in English;
- publication year = 2003+;
- target group must not exclude people age 50+;
- target group must reside in a developed and predominantly western-culture country (North American, antipodean or European continent outside of Russian Federation and Turkey);

In addition, an implemented, evaluated and complete intervention programme that must report outcomes, thus precluding papers that merely identified associations, barriers, needs or ideas about variations in usual practice. Examples of typical outcomes were medication adherence, increase in physical activity, self-monitoring activities (such as blood glucose levels for diabetics), and increases in disease knowledge.

### Screening and further selection

Entries were screened independently by two reviewers (JB and AM, BG or MS) on title and abstract for inclusion criteria, with disagreements resolved upon referral to a third screener (TK) or by discussion. Most review or policy documents which had specific relevant keywords were also hand searched for additional intervention studies that met the inclusion criteria.

The research objective was to choose a relatively small number (<20) of the most promising (and relevant to European priorities) HL intervention programmes to form the basis for practical adaptation and trialling in European populations. Key relevant European public health priorities are many, including: to develop innovative interventions to promote adoption of healthy lifestyles [20, 21], to redress health inequalities between social groups [22, 23], to recommend practical policies and programmes with best evidence of efficacy [21, 24], and to increase lifespans and promote healthy ageing, particularly by addressing the needs of people living with chronic disease [25].

Hence, from our initial inventory of all HL interventions, we identified (from screening title and abstracts) those articles that addressed the most important (in terms of causes of premature death or disability in Europe) diseases or risk factors [26], and that targeted or reported positive changes for vulnerable sub-groups (see item Additional file 1: S5). Next, interventions were assessed by reading the full text of each article and using an 8-point multi-criteria scoring form (MCSF; validity of MCSF methodology is documented in [27]) devised by the authors to further distinguish the most promising and effective interventions that addressed as many aspects of these European public health priorities as possible. Our MCSF questions with instructions are in supplemental files (item Additional file 1: S5). The MCSF questions allowed for a balance of European public health priorities, including: Innovation (see question 1); evidence of testing among vulnerable groups (questions 2 and 3); evidence of improvements in knowledge, behaviour or patient well-being (questions 4, 5 and 6); duration of impacts (question 7); and, quality of study design and evidence (question 8). Each individual question was given a score of 1 (yes) or 0 (no). Total scores were given to each article by at least two independent readers who had read the full article. The scorers were the authors and Irohla partners (see acknowledgements). A final total MCSF score was determined for each article by resolving disagreements via discussion or with reference to a third scorer. Interventions with final scores above five were further evaluated for breadth of strategies they adopted to improve HL, including use of relatively innovative or new methods, as identified by the Irohla model of health literacy [17, 19]. Hence, in addition to the articles which had the highest multi-criteria scores, final selections were made because of included breadth of strategies employed or rarity of that strategy (hence possible innovation), or those items that best targeted health professionals who are important but relatively rare targets for health literacy interventions.

No ethics approval was required for this systematic survey. Statements regarding ethics approval were clearly stated in most if not all of our selected articles.

## Results

### Selection procedure

**Fig. 1:**
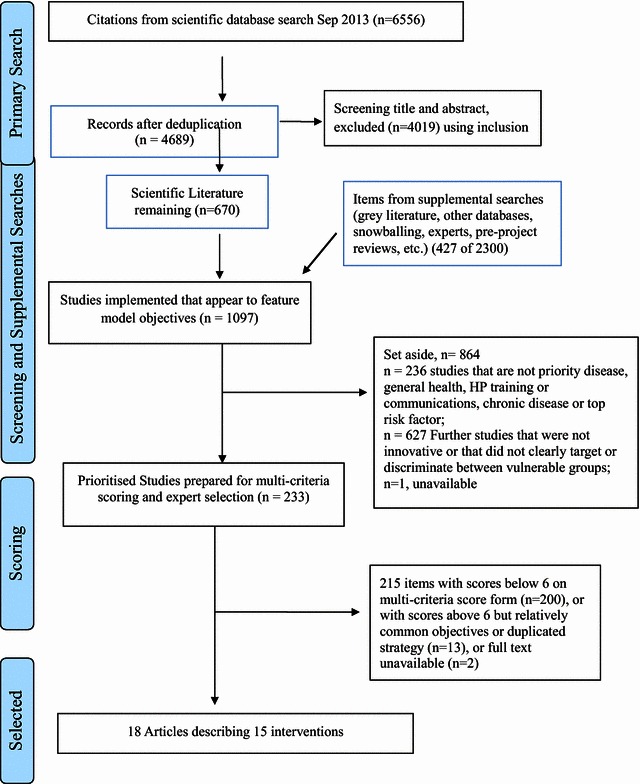
Flow diagram of study selection

In total, 6989 intervention descriptions were found from combined sources (with a small number of duplicates between different types of sources). Most articles (5561 unique reports) came from the scientific literature search. Snowballing revealed a further 421 items in review and policy documents, 371 items came from the English-language Internet search. An additional 53 items were found from the specific searches for articles about disadvantaged European ethnic minorities. Over 500 articles were found and screened from all other sources. Figure 1 shows the selection procedure in (PRISMA style [28]) flow chart format with 1097 items meeting initial inclusion criteria; a PRISMA checklist is available in Additional file 1: Table S6. The main reasons for exclusion were lack of testing on older adults, targets in an ineligible country or, not relevant to health literacy. A total of 233 items were shortlisted for multi-criteria scoring on full text because they featured priority risk factors or diseases, and targeted vulnerable subgroups. The distribution of multi-criteria scores is shown in Additional file 1: Figure S7. Ultimately, the threshold point for choosing the highest scoring programmes was decided based on the distribution of actual scores achieved and the project goal to select only a small number of the most balanced interventions for future testing. More than thirty articles had scores of 6 or above on the multi-criteria scoring form. Seven articles, describing six different programmes, scored 7 or higher. These highest scoring articles were prioritised and supplemented with other interventions to produce a final list of promising interventions that met the comprehensive range of objectives detailed in the IIMHL.

It was desirable that the final chosen programmes offer diverse strategies and address a broad range of diseases and risk factors to ensure study findings have relevance across individual EU countries and communities. After multi-criteria scoring, 15 intervention programmes described in 18 articles were selected as having the best balance of desirable traits, because they demonstrated efficacy among vulnerable target sub-groups, innovative approach to improving HL, greater duration or significance of impacts. These interventions either had the highest scores (multi-criteria score = 7 or greater, n = 7) or at least one of the following traits: a high breadth of intervention strategies (n = 5), especially uncommon strategies (n = 9), possessed the highest multi-criteria scores of those articles concerned with communication with professionals (n = 2) and/or focused on prevention of disease (n = 7). Table 2 summarises the frequency of which strategies were adopted and some target information, while Table 3 lists the final selected interventions.

**Table 2:** Intervention strategies and number of final included articles targeted at each group

Strategy featured, from IIMHL [17]	No. targeted at older adults	No. targeted at health professionals
1 = To inform and educate	14	4
2 = To teach skills	13	4
3 = To support behaviour change and maintenance	15	3
4 = To strengthen contextual support	7	0
5 = To facilitate involvement of individuals at the system level	6	1
6 = To customize HL interventions or enhance their implementation	8	1
7 = To change the social, cultural or physical environment in order to enhance the effects of HL interventions	5	0

**Table 3:** Interventions prioritised for testing in European settings

Title(s) for each unique project or intervention programme	Disease, risk or communication factor	Design and outcomes	IIMHL strategy Nos.	MCS
A randomized trial to improve patient-centered care and hypertension control in underserved primary care patients [33]	Hypertension; Comms with HPs	2 arm RCT study design, physician and patient communication skills both improved significantly, but reductions in blood pressure and changes in adherence were negligible	1,2,3,6, 1p, 2p, 3p, 6p	8
Effects of self-management support on structure, process, and outcomes among vulnerable patients with diabetes [37]; Seeing in 3-D: examining the reach of diabetes self-management support strategies in a public health care system [36]	Diabetes	3 arm RCT, weekly telephone automated support led to more improvements in self management behaviour (SMB) than individual monthly visits from health educator; both improved in SMB over usual care group, but negligible inter-arm differences in clinical indicators	1, 2, 3, 5	7 8
A patient-centric, provider-assisted diabetes tele health self-management intervention for urban minorities [41]	Diabetes	2 arm RCT to test action plan creation with online self-management monitoring, education and group support. Significant improvement in clinical indicators for intervention arm	1, 2, 3, 4	7
Multisite randomized trial of a single-session versus multisession literacy-sensitive self-care intervention for patients with heart failure [34]	CVD	2 arm RCT to test literacy-level appropriate education with ongoing telephone support. Those with lower-literacy had reduced although not significantly lower hospitalisations and rate of death	1, 2, 3	7
I’m taking charge of my arthritis”: designing a targeted self-management program for frail seniors” [29]	MSD	6 home visits to housebound seniors (no controls) to improve self-management. High satisfaction reported by coaches and majority of patients reported significant improvements in psychological well-being and self-efficacy	1, 2, 3, 4	7
Activ-ins-Alter, Active Ageing [32]	Non-specific	No controls. Series of home visits by health counsellors to socioeconomically marginalised people, success was measured with respect to increased knowledge and skills as well as increased willingness to engage with public services and find health information	1, 2, 3, 4, 6, 7	6.5
The ‘10 Keys’ to Healthy Aging: 24-month follow-up results from an innovative community-based prevention program [42]	Non-specific	No controls. Series of home visits by health counsellors to give advice on healthy lifestyle choices, with goal-setting agreed by participants. Resulted in moderate increases in adherence and preventative behaviours	1, 3	6.5
Effectiveness of the chronic disease self-management program for persons with a serious mental illness: a translation study [44] Randomised controlled trial of a lay-led self-management programme for Bangladeshi patients with chronic disease [30]	Depression Any chronic illness	Both Lorig et al. (no controls) and Griffiths et al. (2 arm RCT) are adaptations of same programme for self-management of chronic disease. Composed of 6–8 group support and education sessions led by qualified peer-leaders (culturally sensitive, leaders have same health condition). Main outcomes are significant improvements in many aspects of self-efficacy and perceived well-being either for cohort after 6 months or for intervention arm over controls	1, 2, 3, 4, 5, 6, 7	6.5 6
Developing and testing lay literature about breast cancer screening for African American women [45]; Delta project: increasing breast cancer screening among rural minority and older women by targeting rural healthcare providers [46]	Breast cancer	Comparison group but not RCT. Combination of group counselling and raising physician awareness with custom-designed culturally sensitive literature to inform patients about prevention and detection for breast cancer and to help professionals better counsel patients. Mammogram rates in intervention counties were higher than in comparison counties	1, 2, 3, 4, 5, 6, 7, 1p, 2p, 3p	7 5.5
Promoting a breast cancer screening clinic for underserved women: a community collaboration [38]	Breast cancer	No controls or before/after comparison. Culturally sensitive outreach clinic to underserved minorities to promote sonograms and mammograms. One breast cancer case detected and high satisfaction rates achieved	1, 2, 3, 6, 7	6
Disease management to promote blood pressure control among African Americans [43]	Hypertension	2 arm RCT, lifestyle telephone nurse counselling with educational materials vs. BP monitor alone (control). Intervention group had significant improvements in clinical outcomes and frequency of self-monitoring	1, 2, 3, 6, 1p, 2p	6
Impact of a cardiovascular risk control project for South Asians (Khush Dil) on motivation, behaviour, obesity, blood pressure and lipids [31]	CVD, diabetes, exercise, nutrition	No controls. Small number of targeted culturally sensitive individual counselling sessions with followup and culturally adapted materials. Significant improvement in some lifestyle and diet behaviours	1, 2, 3, 4, 6, 7	6
An advance directive redesigned to meet the literacy level of most adults: a randomized trial [35]	Non-specific	2 arm RCT, testing low literacy vs. standard advance care planning forms. The low-literacy form was preferred by participants and had higher completion rates	1, 3, 5, 6	6
Activating community health centre patients in developing question-formulation skills: a qualitative study [39]	Comms with HPs	No controls. Patients were coached to take an active role and ask assertive questions during medical appointments. Afterwards, most said the preparation was helpful and many noted that they were more actively involved in their appointment than usual	2, 3, 4, 5	4.5
Cancer risk communication with low health literacy patients: a continuing medical education program [40]	Comms with HPs	Cluster RCT. Physicians received training in better communication with low literacy patients and visits from ‘standardised patients’ to assess performance. Negligible differences in physician communication skills between arms	1, 2, 3, 4, 5, 1p, 2p, 3p, 5p	3.5

p suffix denotes strategies targeted at health professionals (HPs)

*MSC* multi-criteria score, *MSD* Musculoskeletal disorders, *CVD* cardiovascular or ischemic heart disease

### Characteristics of selected studies

Most of the intervention participants were in urban communities of the United States. Other interventions took place in Canada [29], the United Kingdom [30, 31] and Austria [32]. The selected interventions address a range of diseases and risk factors, but particularly type 2 diabetes, living with chronic illness, hypertension or cardiovascular disease, and healthy lifestyle changes. Five projects (in six reports) were randomised controlled trials [30, 33–37]. The rest were cohort studies. Most articles tested for statistically significant differences between intervention arms or improvements before and after intervention, and most had modest but statistically significant improvements in aspects of health literacy, such as health knowledge, skills, self-efficacy, quality of communication with health professionals or quality of life outcomes. Six articles [30, 34, 35, 38–40] did not report statistically significant intervention improvements in health-literacy or patient wellbeing outcomes, but were able to cite other benefits such as increased patient satisfaction. Most interventions involved multiple incidences of delivery (i.e., two or more contact sessions with health professionals), but two interventions were single occasion delivery only [35, 39]. Follow-up measurements were taken between 20 min and 24 months post-intervention. The most common intervention format (five programmes in six papers: [29, 34, 36, 37, 41–43]) was an intensive and individualised support programme. These interventions were especially telephone-based (telemedicine), to help people with limited literacy better manage chronic disease or risk factors such as obesity, poor nutrition or lack of physical activity. The chronic disease self-management programme also features twice, in both the United States and the United Kingdom [30, 44]; this is a short series of group sessions to help people manage chronic illness, led by qualified peer support lay health leaders.

Two papers [31, 32] describe community outreach programmes to promote overall active ageing, particularly among vulnerable cultural minorities. Sudore et al. [35] and Pruthi et al. [38] describe low literacy approaches to aid decision-making, and reduce decision conflict, about breast cancer screening and end of life care. Cooper et al. [33] and Lu et al. [39] present action plans to foster better self-assertiveness skills among patients when communicating with health professionals, about either cancer screening or general health questions. Medical staff received individualised feedback and guidance about improving their consultations skills via the mechanisms of video-monitoring and consultations with standardised patients in three projects described in four articles [33, 40, 45, 46]. Pruthi et al. [38] describes community action initiatives to increase awareness and action (to increase mammography screening rates). Together, these 15 programmes described in 18 articles offer a range of diverse strategies and approaches to improving health literacy in older adults.

## Discussion

From almost 7000 studies in many western-culture countries, 15 programmes were identified with promising attributes that could be used as guidance for developing innovative intervention programmes to trial on European older adults. The selected items have multiple positive elements. They provide evidence of efficacy in addressing high priority diseases and risk factors as well as the public health policy goal of disease prevention. They describe success in patient groups that are vulnerable or in health professionals trying to address a clinical risk factor. The selected interventions present a wide breadth of health promotion strategies and address diverse health problems. The interventions collected through this comprehensive and systematic survey are supported by multi-cultural theoretical and practical health promotion frameworks [47–49].

Virtually all interventions which scored highly in the multi-criteria scoring exercise aimed to educate, improve patient skills and motivate. Cultural adaptations and actions to change the physical environment (such as paying for travel expenses) were also common. Although target groups in the final interventions are both older adults and professionals, health professionals are much less often the targets of health literacy studies in well-evaluated and documented research, although health literacy often features in professional training [50]. Only six of the final interventions featured the involvement of patients at a systems level (strategy No. 5 in Table 2); previous research showed this to be the rarest strategy in HL interventions [17]. Yet, this particular strategy may be the most crucial to reducing the deleterious impacts that poor health literacy has upon population health outcomes. We lack space to fully explore the relevant debates, but it is worth noting that health literacy is not always seen as an individual responsibility. Salter et al. (2014) explored the heavy health literacy burden that modern health care systems impose particularly on older adults with chronic illness. Hence, an emphasis in public health programmes on individual health literacy may occur at the expense of ignoring the social determinants of health [51] and poor HL may be seen as a result of poor health care delivery [52]. It has even been argued that health literacy interventions would be better viewed as treatment rather than education [53]. There is also evidence to suggest that at least some interventions, even those in randomised controlled trial format, have negligible impacts and relevance due to poor planning before and insufficient process evaluation after implementation [54, 55].

For now, building on the search and grading work described here, the Irohla project intends to recommend interventions that are most likely to be successful for older adults in Europe. Recommendations will combine the replication of some interventions previously shown as successful, but with further evidence for suitable social and cultural adaptations [19].

### Strengths and limitations

We do not know of a similar inventory to the one which we created, or of a similar effort to assess suitability from so many intervention programmes to identify a small number that are best for trial in the European context. Our approach was necessarily driven by the practical needs and objectives of the funders to promote healthy ageing. The diversity of selected interventions, in terms of implementation plans, delivery modes and frequency, as well as duration of measured impacts, is valuable. To be readily adaptable for culturally diverse European populations with increasingly complex health needs and risk factors due to ageing demographics [56, 57], health literacy interventions recommended by Irohla need to be flexible and multi-faceted.

Inevitably, the selection process was adapted to the data available and to the demands of the development process needed to inform future practical EU guidance. Search terms were necessarily limited and meant, for instance, that relatively few studies were found about some widely used communication methods such as Teach Back [58, 59]. We lacked resources to hand search all review documents and had to prioritise those with relevant key words about target groups or conditions. The search was reliant on indexing conventions in scientific databases as well as the content of abstracts and titles; as a result inadequate reporting in some abstracts [60] means that some promising interventions may have been missed. However, we attempted to overcome this by seeking expert opinion, checking reference lists of other papers, searching for national guidelines etc.

Response from the expert survey (to obtain information on tested but unpublished interventions) was low. Non English-language Internet results were generally less useful than those available in the English language. Importantly, the phrase and concept health literacy has not been widely adopted by European public health agencies which led to some subjectivity when screening and applying eligibility criteria. Ethnic minorities specific to the United States (Hispanics and African Americans) were frequent participants in the selected interventions; one of the subsequent challenges for Irohla will be how to appropriately adapt the principles of these successful programmes for use among European ethnic and minority groups.

Our multi-criteria scoring was probably weighted in favour of studies with demonstrable improvements in outcomes rather than type of evidence, duration of interventions or innovation. This may be a strength or a limitation of our work (depending on what priorities are foremost in mind of the reader). We were also not able to rigorously evaluate innovative but untested intervention ideas.

## Conclusion

From thousands of intervention studies, we identified 15 promising programmes that featured strong evidence of efficacy among important diseases or risk factors and vulnerable groups, or that had some evidence of success and important innovation in older populations. To narrow down such a large group of items to such a small selection was challenging and the choices may never be perfect, but they should suggest a broad range of evidence-based approaches for future intervention development and testing on the diverse European continent. HL interventions that work elsewhere may not be able to deliver similar results among European populations. The interventions that we have prioritized are those with (some) evidence of efficacy, targeted at vulnerable groups with health conditions important to the European older adult population.

Unfortunately, health literacy interventions tend to lack rigorous study design [61, 62] and therefore it is premature [62] to try to select the very best health literacy interventions Nevertheless, five of our final selected projects featured the highest standard of clinical evidence of possible success (i.e., from a randomised control trial, or RCT). More clearly-reported research in the RCT format would be welcome. The observed potential lack of compelling evidence presents an opportunity to test both established and innovative ideas under a robust study design.

## Additional file

10.1186/s13104-016-2056-9 Irohla model taxonomy, search strategy details, multi-criteria scoring sheet and results, and PRISMA checklist.

## Abbreviations

CINAHL: cumulative index to nursing and allied health literature
EU: European union
HL: health literacy
IIMHL: Irohla intervention model of health literacy
Irohla: intervention research on health literacy in ageing populations
MCSF: multi-criteria scoring form
RCT: randomised controlled trial

## References

[1] Kwan B, Frankish J, Rootman I, Zumbo B, Kelly K, Begoray D, Kazanijan A, Mullet J, Hayes M. The development and validation of measures of “health literacy” in different populations. UBC Institute of Health Promotion Research and University of Victoria Community Health Promotion Research 2006.

[2] Jochelson K (2007). Health literacy review paper.

[3] Nutbeam D (2008). The evolving concept of health literacy. Social Sci Med.

[4] Peerson A, Saunders M (2009). Health literacy revisited: what do we mean and why does it matter?. Health Promot Int.

[5] Sørensen K, Van den Broucke S, Fullam J, Doyle G, Pelikan J, Slonska Z, Brand H (2012). Health literacy and public health: a systematic review and integration of definitions and models. BMC Public Health.

[6] Salter C, Brainard J, McDaid L, Loke Y (2014). Challenges and opportunities: what can we learn from patients living with chronic musculoskeletal conditions, health professionals and carers about the concept of health literacy using qualitative methods of inquiry?. PLoS ONE.

[7] Nutbeam D (2000). Health literacy as a public health goal: a challenge for contemporary health education and communication strategies into the 21st century. Health Promot Int.

[8] ABS: experimental estimates and projections, aboriginal and Torres strait islander Australians, 1991–2021 vol. Cat. No. 3238.0: Australian Bureau of Stastistics; 2009.

[9] HLS-EU Consortium: comparative report of health literacy in eight EU member states. The European Health Literacy Survey HLS-EU; 2012.

[10] Paasche-Orlow MK, Parker RM, Gazmararian JA, Nielsen-Bohlman LT, Rudd RR (2005). The prevalence of limited health literacy. J General Intern Med.

[11] Berkman ND, Sheridan SL, Donahue KE, Halpern DJ, Crotty K (2011). Low health literacy and health outcomes: an updated systematic review. Ann Intern Med.

[12] Eichler K, Wieser S, Brügger U (2009). The costs of limited health literacy: a systematic review. Int J Public Health.

[13] EurActiv: resilient and innovative EU health systems: special report; 2013.

[14] European patients forum: making health literacy a priority in EU policy. http://www.eu-patient.eu/globalassets/policy/healthliteracy/health-literacy-concept-paper_final.pdf. Accessed 12 Dec 2014.

[15] European commission: European citizens’ digital health literacy. 2014: 221.

[16] Bostock S, Steptoe A (2012). Association between low functional health literacy and mortality in older adults: longitudinal cohort study. BMJ.

[17] Irohla consortium: overview of activities and results of WP2 deliverable D2.2; 2013.

[18] Irohla consortium: IROHLA project progress report, community research and development information service. http://cordis.europa.eu/docs/results/305/305831/periodic1-irohla-project-progress-report-public-summary.pdf. Accessed 15 Feb 2016.

[19] IROHLA consortium: IROHLA project progress report: public report June 2014. vol 1; 2014.

[20] Sørensen TIA. Report of the independent expert group on the future of European public health research: European commission; 2013.

[21] European commission. On effective, accessible and resilient health systems. European commission: Brussels; 2014.

[22] European commission: European platform against poverty and social exclusion. http://ec.europa.eu/social/main.jsp?catId=961&langId=en&moreDocuments=yes. Accessed 11 Feb 2015.

[23] Marmot M, Allen J, Bell R, Bloomer E, Goldblatt P (2012). WHO European review of social determinants of health and the health divide. Lancet.

[24] Guest C, Ricciardi W, Kawachi I, Lang I (2013). Oxford handbook of public health practice.

[25] EIP-AHA. European scaling-up strategy in active and healthy ageing; 2014.

[26] World Health Organization. Global health risks: mortality and burden of disease attributable to selected major risks. World Health Organization; 2009.

[27] Husereau D, Boucher M, Noorani H (2010). Priority setting for health technology assessment at CADTH. Int J Technol Assess Health Care.

[28] Liberati A, Altman DG, Tetzlaff J, Mulrow C, Gøtzsche PC, Ioannidis JP, Clarke M, Devereaux PJ, Kleijnen J, Moher D (2009). The PRISMA statement for reporting systematic reviews and meta-analyses of studies that evaluate health care interventions: explanation and elaboration. Ann Intern Med.

[29] Laforest S, Nour K, Parisien M, Poirier M-C, Gignac M, Lankoande H (2008). “I’m Taking Charge of My Arthritis”: designing a targeted self-management program for frail seniors. Phys Occup Ther Geriatr.

[30] Griffiths C, Motlib J, Azad A, Ramsay J, Eldridge S, Feder G, Khanam R, Munni R, Garrett M, Turner A (2005). Randomised controlled trial of a lay-led self-management programme for Bangladeshi patients with chronic disease. Br J General Prac.

[31] Mathews G, Alexander J, Rahemtulla T, Bhopal R (2007). Impact of a cardiovascular risk control project for South Asians (Khush Dil) on motivation, behaviour, obesity, blood pressure and lipids. J Public Health.

[32] Active ageing. Aktiv ins Alter! Investition in die Gesundheit älterer Menschen; 2008.

[33] Cooper LA, Roter DL, Carson KA, Bone LR, Larson SM, Miller ER, Barr MS, Levine DM (2011). A randomized trial to improve patient-centered care and hypertension control in underserved primary care patients. J General Intern Med.

[34] DeWalt DA, Schillinger D, Ruo B, Bibbins-Domingo K, Baker DW, Holmes GM, Weinberger M, Macabasco-O’Connell A, Broucksou K, Hawk V. A multisite randomized trial of a single-versus multi-session literacy sensitive self-care intervention for patients with heart failure. Circulation 2012. doi:10.1161/CIRCULATIONAHA.111.081745. 10.1161/CIRCULATIONAHA.111.081745 PMC3400336 22572916

[35] Sudore RL, Landefeld CS, Barnes DE, Lindquist K, Williams BA, Brody R, Schillinger D (2007). An advance directive redesigned to meet the literacy level of most adults: a randomized trial. Patient Educ Counsel.

[36] Schillinger D, Hammer H, Wang F, Palacios J, McLean I, Tang A, Youmans S, Handley M (2008). Seeing in 3-D: examining the reach of diabetes self-management support strategies in a public health care system. Health Educ Behav.

[37] Schillinger D, Handley M, Wang F, Hammer H (2009). Effects of self-management support on structure, process, and outcomes among vulnerable patients with diabetes a three-arm practical clinical trial. Diabetes Care.

[38] Pruthi S, Shmidt E, Sherman MM, Neal L, Wahner-Roedler D (2010). Promoting a breast cancer screening clinic for underserved women: a community collaboration. Ethn Dis.

[39] Lu W-H, Deen D, Rothstein D, Santana L, Gold MR. Activating community health center patients in developing question-formulation skills: a qualitative study. Health Educ Behav. 2011: 1090198110393337. 10.1177/1090198110393337 21558464

[40] Price-Haywood EG, Roth KG, Shelby K, Cooper LA (2010). Cancer risk communication with low health literacy patients: a continuing medical education program. J General Intern Med.

[41] Carter EL, Nunlee-Bland G, Callender C. A patient-centric, provider-assisted diabetes tele health self-management intervention for urban minorities. Perspectives in health information management/AHIMA, American health information management association 2011, 8(Winter). PMC3035826 21307985

[42] Robare JF, Bayles C, Newman AB, Williams K, Milas C, Boudreau R,  McTigue K, Albert SM, Taylor C, Kuller LH (2010). The “10 Keys” to healthy aging: 24-month follow-up results from an innovative community-based prevention program. Health Educ Behav.

[43] Brennan T, Spettell C, Villagra V, Ofili E, McMahill-Walraven C, Lowy EJ, Daniels P, Quarshie A, Mayberry R (2010). Disease management to promote blood pressure control among African Americans. Population Health Manag.

[44] Lorig K, Ritter PL, Pifer C, Werner P (2014). Effectiveness of the chronic disease self-management program for persons with a serious mental illness: a translation study. Commun Mental Health J.

[45] Coleman EA, Coon S, Mohrmann C, Hardin S, Stewart B, Gibson RS, Cantrell M, Lord J, Heard J (2003). Developing and testing lay literature about breast cancer screening for African American women. Clin J Oncol Nurs.

[46] Coleman EA, Lord J, Heard J, Coon S, Cantrell M, Mohrmann C, O’Sullivan P. The Delta project: increasing breast cancer screening among rural minority and older women by targeting rural healthcare providers. In: Oncology nursing forum 2003. Onc Nurs Soc. 2003. 10.1188/03.ONF.669-677 12861326

[47] Dadaczynski K, Paulus P, de Vries N, de Ruiter S, Buijs G. HEPS inventory tool: an Inventory tool including quality assessment of school interventions on healthy eating and physical activity. Online Submission 2010.

[48] National collaborating centre for methods and tools tool for grading public health interventions (NICE Tool): Hamilton: McMaster University; 2009.

[49] BZgA/UKE Med. Psych: an evidence-based quality assessment tool for prevention and health promotion activities. vol 2003–2010. BZgA; 2011.

[50] Irohla consortium. Overview of activities and results of WP4 deliverable D4.4; 2014.

[51] Volandes AE, Paasche-Orlow MK (2007). Health literacy, health inequality and a just healthcare system. Am J Bioethic.

[52] Raynor DK (2012). Health Literacy. BMJ.

[53] McCallum A. Director of public health and public policy, NHS lothian. 2014. Personal communication to Brainard J. Nov 22.

[54] Bornhöft G, Maxion-Bergemann S, Wolf U, Kienle GS, Michalsen A, Vollmar HC, Gilbertson S, Matthiessen PF (2006). Checklist for the qualitative evaluation of clinical studies with particular focus on external validity and model validity. BMC Med Res Methodol.

[55] Murdoch J, Varley A, Lattimer V, Campbell J, Salter C. Designing process evaluations for cluster randomised controlled trials: definitions of context and implications for data collection. In: North American primary care group annual meeting: 21–25 November 2014; New York.

[56] Börsch-Supan A, Hank K, Jürges H (2005). A new comprehensive and international view on ageing: introducing the ‘survey of health, ageing and retirement in Europe’. Eur J Age.

[57] Christensen K, Doblhammer G, Rau R, Vaupel JW (2009). Ageing populations: the challenges ahead. Lancet.

[58] Dinh HTT, Clark R, Bonner A, Hines S (2013). The effectiveness of health education using the teach-back method on adherence and self-management in chronic disease: a systematic review protocol. JBI Database Syst Rev Implemen Rep.

[59] White M, Garbez R, Carroll M, Brinker E, Howie-Esquivel J (2013). Is “Teach-Back” associated with knowledge retention and hospital readmission in hospitalized heart failure patients?. J Cardiovasc Nurs.

[60] Brainard J, Loke Y, Salter C. International variations in targeting of vulnerable groups: health literacy interventions. In: European public health association meeting: Nov 22 2014; Glasgow.

[61] Brainard J, Howard-Wilsher S, Loke Y, Salter C. Quality and efficacy of health literacy randomised controlled trials. (Under review).

[62] Heijmans M, Uiters E, Rose T, Hofstede J, Devillé W, van der Heide I, Boshuisen H, Rademakers J (2015). Study on sound evidence for better understanding of health literacy in the European Union.

