# Supplementary material for: Healthy ageing in Europe: prioritizing interventions to improve health literacy
Source: BMC Res Notes. 2016 May 12;9:270. doi: 10.1186/s13104-016-2056-9 (PMC4866482; doi:10.1186/s13104-016-2056-9)
Supplement: Supplementary file 1 — 10.1186/s13104-016-2056-9 Irohla model taxonomy, search strategy details, multi-criteria scoring sheet and results, and PRISMA checklist. [file 13104_2016_2056_MOESM1_ESM.docx]

**Table S1** IROHLA taxonomy for health interventions [[1-3](#_ENREF_1)]

|  | **Objectives** | **Activities** | **Examples** |
| --- | --- | --- | --- |
| **1** | Inform and educate | Provide easily accessible and  understandable information in plain language | Resdesign information materials; use innovative communication. Develop easily accessible websites or apps. Integrate health literacy into adult literacy programmes. Sensitise professionals about health literacy and their role in patient support. |
| **2** | Teach skills | Teach people how to handle  concrete situations in real life | Develop computer literacy among older people. Instruct them how to use e-health apps. Train older people and professionals in communication, asking the right questions, testing whether answers were understood. |
| **3** | Support behavioural  change and maintain  it over time | Help people to acquire a positive attitude to overcoming health literacy challenges and keep it up over time | Changing behaviour takes time: ensure follow up, e.g. telephone, home visits, community clubs, or virtual communities. Motivate older people, and also health care professionals in (virtual) communities. |
| **4** | Strengthen  contextual support | Mobilise and empower relatives,  friends, communities to support  persons with health literacy needs | Train caregivers in understanding and handling the health issues of older people. Organise clubs or meeting places for social contacts among older people. Facilitate mutual support groups. |
| **5** | Facilitate  involvement of  individuals at the system level | Give people a voice in changing  organisations and in addressing  relevant issues for improvement of health literacy | Develop e-health together with users. Develop training courses with the future clients. Use patient panels to give feedback on performance of health institutions and to give suggestions. |
| **6** | Customise or enhance intervention | Adjust implementation to increase chances of intervention success | Provide cues-to action, or appointment reminders.  Increase duration of consultations with individuals who have low health literacy. |
| **7** | Change the environment to enhance impact | Change the social, cultural or  physical environment and make it easier for people to obtain access to the support they need | Make signboards and (electronic) appointment systems in hospitals easy to understand. Give tablets or iPads for communication in homecare. Provide transport. |

Box S2. Expert, specific database, snowballing and other search methods

- Most review and policy documents were searched by hand if they contained relevant keywords (eg older adults, health literacy)
- EIP-AHA [[4](#_ENREF_4), [5](#_ENREF_5)] adherence best practice booklet: the phrases older and literacy were used to find interventions. EIP-AHA marketplace initiative: all 99 items within the “health literacy” pillar were searched for relevance
- BZgA [[6](#_ENREF_6)] healthy and active ageing compendium 2012: searched by hand
- RIVM [[7](#_ENREF_7)] : searched (all types of document) using the terms “health literacy” or “health promotion”
- NICE [[8](#_ENREF_8)] : searched using the phrase ( (“health literacy” or “adherence or self-management) AND (“older adults” or “senior citizens”) )
- EPHA [[9](#_ENREF_9)] : some review of website; their conference events are usually indexed in scientific literature
- IUHPE [[10](#_ENREF_10)] : some review of website; articles in the organisation’s official journal and associated journals are indexed in OVID, CINAHL, etc.
- EUPHA [[11](#_ENREF_11)] : some review of website; articles in the organisation’s official journal is indexed in OVID, CINAHL, etc.
- PROGRESS [[12](#_ENREF_12)] : searched on website for health literacy
- Expert recommendations: Invited all experts in project Delphi database to take an online survey, to especially identify unpublished research
- healthPROelderly [[13](#_ENREF_13)] : searched among 174 projects for lifelong/learning education theme, with Targets = disadvantaged, handicapped, minority ethnic or migrants, excluding Lifestyle factor=violence
- RTIPS (NCI) [[14](#_ENREF_14)] :searched among 143 programmes for target disease or risk factors, mentioning adults age 40+, not taking place in school or preschool venues
- Results from pre-project searches and items otherwise known to partners: were also added to the databases in appropriate categories (such as scientific literature, Internet, etc.)

**Acronyms:**

BZgA: Bundeszentrale für gesundheitliche Aufklärung (The Federal Centre for Health Education)

EIP-AHA: European Innovative Partnerships - Healthy and Active Ageing

EPHA: European Public Health Alliance

EUPHA: European Public Health Association

IUPHE: [International Union for Health Promotion and Education](http://www.iuhpe.org/)

NICE: National Institute for Health and Care Excellence

PROGRESS: Progress towards healthy ageing in europe

RIVM: [Rijksinstituut voor Volksgezondheid en Milieu](http://www.rivm.nl/RIVM) (Netherlands National Institute for Public Health and the Environment:)

RTIPS (NCI): Research Tested Innovation Programs (National Cancer Institute)

Box S3: Search phrases used in Google Advanced Search to find Internet and Grey literature

| - find and understand health related information AND interventions AND (older-adults OR seniors OR retired OR pensioners OR aging-population OR elderly OR aged or family OR community OR geriatric intention) OR (motivation OR adoption OR promotion OR self-efficacy OR personal-goal-setting OR maintenance OR behavioural-change) |
| --- |
| - management of disease programs AND (older adults OR seniors OR retired OR pensioners OR aging-population OR elderly) AND health literacy interventions - self-management in health AND (Older adults OR seniors OR retired OR pensioners OR aging-population OR elderly) AND "health literacy interventions for older adults" |
| - adherence to medication programs AND (older OR adults OR seniors OR retired OR pensioners OR aging-population) AND health literacy interventions elderly seniors |
| - interventions communication of health professionals AND (older-adults OR seniors OR retired pensioners OR aging-population OR elderly OR carers) AND (Strategies OR programs OR campaigns OR Interventions OR activities OR examples OR practices OR innovations OR health OR promotion OR prevention OR "informed decision making") |
| - interventions communication of health professionals AND (older-adults OR seniors OR retired pensioners OR aging-population OR elderly OR carers) AND (Strategies OR programs OR campaigns OR Interventions OR activities OR examples OR practices OR innovations OR health OR promotion OR prevention "Quality of communication") |
| - interventions skills of health professionals AND (older-adults OR seniors OR retired pensioners OR aging-population OR elderly OR carers) AND (Strategies OR programs OR campaigns OR Interventions OR activities OR examples OR practices OR innovations OR health promotion OR prevention "provide information ") |
| - health professionals AND (older-adults OR seniors OR retired pensioners OR aging-population OR elderly OR carers awareness) OR knowledge OR "Doctor patient communication" |
| - intervention AND (older adults OR seniors OR pensioners OR elderly OR older consumers OR geriatric) AND (social OR marketing OR pensions OR insurance) AND "numeracy literacy" |
| - health professionals AND (older-adults OR seniors OR retired pensioners OR aging-population OR elderly OR carers) AND (awareness OR knowledge "health-literacy interventions") |

**Box S4.** Keywords used to prioritise hand searches for further references in systematic and other types of literature reviews from peer-review or grey literature, as well as policy documents or reports in grey literature:

Older adults, seniors, aged, age 50+, elderly, frail, elders, senior citizens, ethnic, health literacy, European minorities

**Supplemental Item S5. MULTI-CRITERIA SCORE SHEET INSTRUCTIONS**

The questions are to help determine how well this intervention fits with the IROHLA objectives:

Question 1:

In view of the taxonomy in the IROHLA model of health literacy (consult irohla deliverable 2.2), interventions may be judged ‘Innovative’ if they have at least one of the following features:

1. ***Uncommon Strategies.*** Some strategies were seldom included in previous interventions, and thus might be considered as more innovative. These strategies feature at least one of the following –

*******strengthening contextual support,

*******facilitating involvement of individuals at the system level,

*******customizing health literacy interventions,

*******enhancing the implementation of these interventions, **and/or**

*******changing the social, cultural or physical environment in order to enhance the effects of health literacy interventions.

1. ***Targets multiple aspects of health literacy***. This includes broad, multi-faceted approaches that involve both older adults, professionals and their context through a few different strategies.
2. ***Innovative strategies***. Use of technology, peer education or an otherwise unusual approach.

Questions 2 + 3 (Target Groups):

Must be very clearly stated to score 1.

Score Yes if interventions were targeted at individuals with a low health literacy or socioeconomic status (e.g. level of education, income, mention of social deprivation), or at individuals living in communities with these traits as noted by the intervention description. Other low SE indicators might be a high percentage of adults with low educational attainment, high unemployment rates or a labour force composed predominantly of low-wage earners.

Question 4. Specific health literacy outcomes:

Score yes if there are positive changes in any of these: understanding health documents, knowledge, self-efficacy, making informed decisions, self-management skills or social engagement.

Question 5. Intermediate outcomes:Score yes if there are positive changes in any of these: health behaviours, motivation, access and participation in health care.

Question 6: Improvement in Indicators of patient health or well-being**.**  This is about the main health outcomes that patients and clinicians will be most aware of and tend to be final rather than intermediate, such as reduction in anxiety or pain, or decreases in risk factors such as weight loss, quality of life, better function etc.

Question 7: Apply to any positive outcome measure, intermediate, final or otherwise. To score yes, a positive outcome must be reported at least six months after the intervention programme finished.

Question 8: Must be very clearly stated to score yes.

Question 9: Other comments that Scorer feels are relevant.

Comments: If convenient, in this column, please note which page of an article substantiates the answer, or copy and paste text; especially for high scoring intervention descriptions (total score >=6). Other comments are fine, too.

**SCORING:** All questions are worth either 0 or 1 point. If in doubt, score conservatively (0 rather than 1) Add up scores and total at bottom.

**MULTI-CRITERIA SCORE SHEET**

Title of Intervention Description + author (s):

Reviewer Name:

Other comments?

With regards to this particular intervention, please use your expert judgment to answer every question, Score 1 = Yes, Score 0 = No.

|  | Score | Comments |
| --- | --- | --- |
| 1. Is the intervention innovative? |  |  |
| 2. Are the participants identified as older adults who have low literacy/HL, or who are from a community that is at high risk of low Literacy/HL? |  |  |
| 3. Has the intervention been tested on older adults with low socio-economic status or who are at high risk of low SES? |  |  |
| 4. Health Literacy outcomes: Does the evaluation report positive impacts on at least one health literacy outcome? |  |  |
| 5. Intermediate outcomes: Does the evaluation report positive impacts on at least one intermediate outcome? |  |  |
| 6. Health outcomes: Does the evaluation report positive impacts on at least one measure of participant health or well-being? |  |  |
| 7. Does the intervention show benefits or at least one positive outcome at six months post intervention or later? |  |  |
| 8. Do the results come from a Randomized Clinical Trial? |  |  |
| 9. Other comments? |  |  |
| Total Score |  |  |

**Table S6. PRISMA checklist**

| **Section/topic** | | **#** | | **Checklist item** | | **Reported on page #** |
| --- | --- | --- | --- | --- | --- | --- |
| **TITLE** | | | | | |  |
| Title | | 1 | | Identify the report as a systematic review, meta-analysis, or both. | | Not done |
| **ABSTRACT** | | | | | |  |
| Structured summary | | 2 | | Provide a structured summary including, as applicable: background; objectives; data sources; study eligibility criteria, participants, and interventions; study appraisal and synthesis methods; results; limitations; conclusions and implications of key findings; systematic review registration number. | | 2 |
| **INTRODUCTION** | | | | | |  |
| Rationale | | 3 | | Describe the rationale for the review in the context of what is already known. | | 2,3 |
| Objectives | | 4 | | Provide an explicit statement of questions being addressed with reference to participants, interventions, comparisons, outcomes, and study design (PICOS). | | 2-6 |
| **METHODS** | | | | | |  |
| Protocol and registration | | 5 | | Indicate if a review protocol exists, if and where it can be accessed (e.g., Web address), and, if available, provide registration information including registration number. | | Not done |
| Eligibility criteria | | 6 | | Specify study characteristics (e.g., PICOS, length of follow-up) and report characteristics (e.g., years considered, language, publication status) used as criteria for eligibility, giving rationale. | | 2-6 |
| Information sources | | 7 | | Describe all information sources (e.g., databases with dates of coverage, contact with study authors to identify additional studies) in the search and date last searched. | | 4, supplemental |
| Search | | 8 | | Present full electronic search strategy for at least one database, including any limits used, such that it could be repeated. | | 4, supplemental |
| Study selection | | 9 | | State the process for selecting studies (i.e., screening, eligibility, included in systematic review, and, if applicable, included in the meta-analysis). | | 4-6 |
| Data collection process | | 10 | | Describe method of data extraction from reports (e.g., piloted forms, independently, in duplicate) and any processes for obtaining and confirming data from investigators. | | 5-6 |
| Data items | | 11 | | List and define all variables for which data were sought (e.g., PICOS, funding sources) and any assumptions and simplifications made. | | 5-6, supplemental |
| Risk of bias in individual studies | | 12 | | Describe methods used for assessing risk of bias of individual studies (including specification of whether this was done at the study or outcome level), and how this information is to be used in any data synthesis. | | Not done |
| Summary measures | | 13 | | State the principal summary measures (e.g., risk ratio, difference in means). | | Not done |
| Synthesis of results | | 14 | | Describe the methods of handling data and combining results of studies, if done, including measures of consistency (e.g., I^2^) for each meta-analysis. | | Not done |
| **Section/topic** | **#** | | **Checklist item** | | **Reported on page #** | |
| Risk of bias across studies | 15 | | Specify any assessment of risk of bias that may affect the cumulative evidence (e.g., publication bias, selective reporting within studies). | | Not done | |
| Additional analyses | 16 | | Describe methods of additional analyses (e.g., sensitivity or subgroup analyses, meta-regression), if done, indicating which were pre-specified. | | 5-6 (multi-criteria scoring) | |
| **RESULTS** | | | | |  | |
| Study selection | 17 | | Give numbers of studies screened, assessed for eligibility, and included in the review, with reasons for exclusions at each stage, ideally with a flow diagram. | | Separate figure, 6-7 | |
| Study characteristics | 18 | | For each study, present characteristics for which data were extracted (e.g., study size, PICOS, follow-up period) and provide the citations. | | Partly done, Tables 2-3 | |
| Risk of bias within studies | 19 | | Present data on risk of bias of each study and, if available, any outcome level assessment (see item 12). | | Not done | |
| Results of individual studies | 20 | | For all outcomes considered (benefits or harms), present, for each study: (a) simple summary data for each intervention group (b) effect estimates and confidence intervals, ideally with a forest plot. | | Partly Table 3, rest Not applicable | |
| Synthesis of results | 21 | | Present results of each meta-analysis done, including confidence intervals and measures of consistency. | | Not done | |
| Risk of bias across studies | 22 | | Present results of any assessment of risk of bias across studies (see Item 15). | | Not done | |
| Additional analysis | 23 | | Give results of additional analyses, if done (e.g., sensitivity or subgroup analyses, meta-regression [see Item 16]). | | Not done | |
| **DISCUSSION** | | | | |  | |
| Summary of evidence | 24 | | Summarize the main findings including the strength of evidence for each main outcome; consider their relevance to key groups (e.g., healthcare providers, users, and policy makers). | | 8-10 | |
| Limitations | 25 | | Discuss limitations at study and outcome level (e.g., risk of bias), and at review-level (e.g., incomplete retrieval of identified research, reporting bias). | | 9-10 | |
| Conclusions | 26 | | Provide a general interpretation of the results in the context of other evidence, and implications for future research. | | 10 | |
| **FUNDING** | | | | |  | |
| Funding | 27 | | Describe sources of funding for the systematic review and other support (e.g., supply of data); role of funders for the systematic review. | | 1, 3, 11 | |

*From:*  Liberati et al (2009), The PRISMA statement for reporting systematic reviews and meta-analyses of studies that evaluate health care interventions: explanation and elaboration. Annals of Internal Medicine 151(4) 65-94.

For more information, visit: **www.prisma-statement.org**.

Page 3 of 3

**Figure S7.** Multi-criteria scores for interventions in 233 articles subject to full text review.

*Note*: Scores of zero resulted when full text was unavailable or intervention was found to be ineligible upon full text review.

**REFERENCES**

1. Irohla Consortium: **Overview of Activities and Results of WP2 Deliverable D2.2**; 2013.

2. IROHLA Consortium: **IROHLA Project Progress Report: Public Report June 2014**, vol. 1; 2014.

3. IROHLA Consortium: **Policy Brief for Health Organisations**. In*.*: Health Literacy Centre Europe; 2015.

4. EIP-AHA: **European Innovation Partnership on Active and Healthy Aging Action Group A1**, Accessed: November 2013 [[https://webgate.ec.europa.eu/eipaha/library/index/download/id/726]](https://webgate.ec.europa.eu/eipaha/library/index/download/id/726%5d).

5. EIP-AHA: **European Innovation Partnership on Active and Healthy Initiatives**, Accessed: January 2014 [[https://webgate.ec.europa.eu/eipaha/initiative]](https://webgate.ec.europa.eu/eipaha/initiative%5d).

6. BZgA: **Healthy and Active Ageing**. Brussels, Belgium: Bundeszentrale für gesundheitliche Aufklärung, EurohealthNet.

7. Netherlands National Institute for Public Health and the Environment: Rijksinstituut voor Volksgezondheid en Milieu, Accessed: 15 December 2013 [[http://www.rivm.nl/English]](http://www.rivm.nl/English%5d).

8. National Institute for Health and Care Excellence: Accessed: 20 July 2015 [[http://www.nice.org.uk/]](http://www.nice.org.uk/%5d).

9. European Public Health Alliance: Accessed: 20 July 2015 [[http://www.epha.org/]](http://www.epha.org/%5d).

10. International Union for Health Promotion and Education: Accessed: 16 December 2013 [[http://www.iuhpe.org/index.php/en/]](http://www.iuhpe.org/index.php/en/%5d).

11. European Public Health Association: Accessed: [[http://www.eupha.org/]](http://www.eupha.org/%5d).

12. Progress Towards healthy ageing in Europe: Accessed: 20 July 2015 [[http://www.progresshealthyageing.eu/]](http://www.progresshealthyageing.eu/%5d).

13. Health Pro Elderly: Bundesminsterium fur Gesundheit, Accessed: 20 January 2014 [[http://www.healthproelderly.com/]](http://www.healthproelderly.com/%5d).

14. Research Tested Innovation Programs (RTIPS): National Cancer Institute, Accessed: 20 January 2014 [[http://rtips.cancer.gov/rtips/index.do]](http://rtips.cancer.gov/rtips/index.do%5d).
